# Activation and inhibition of posterior parietal cortex have bi-directional effects on spatial errors following interruptions

**DOI:** 10.3389/fnsys.2014.00245

**Published:** 2015-01-12

**Authors:** Cyrus K. Foroughi, Eric J. Blumberg, Raja Parasuraman

**Affiliations:** Department of Psychology, Arch Lab, George Mason UniversityFairfax, VA, USA

**Keywords:** tDCS, brain stimulation, spatial errors, interruptions, posterior parietal cortex, mental rotation, cognitive equalizing

## Abstract

Interruptions to ongoing mental activities are omnipresent in our modern digital world, but the brain networks involved in interrupted performance are not known, nor have the activation of those networks been modulated. Errors following interruptions reflect failures in spatial memory, whose maintenance is supported by a brain network including the right posterior parietal cortex (PPC). The present study therefore used bi-directional transcranial Direct Current Stimulation (tDCS) of right PPC to examine the neuromodulation of spatial errors following interruptions, as well as performance on another PPC-dependent task, mental rotation. Anodal stimulation significantly reduced the number of interruption-based errors and increased mental rotation accuracy whereas cathodal stimulation significantly increased errors and reduced mental rotation accuracy. The results provide evidence for a causal role of the PPC in the maintenance of spatial representations during interrupted task performance.

## Introduction

Interruptions to our ongoing mental activities are omnipresent in modern life—whether from cell phones, emails, navigation devices, alarms, etc. An observational study found that people are interrupted an average of 12 times per hour at work in our increasingly digital world (Cades et al., 2010), with such interruptions often leading to errors. Another study of nurses from two hospitals showed that interruptions increased both procedural (e.g., fail to check patient identification) and clinical judgment errors (e.g., give the wrong drug or wrong dose), with potentially life threatening consequences (Westbrook et al., 2010). Interruption-related errors are ubiquitous and appear to be unrelated to individual expertize (e.g., Dismukes et al., 2012; Prakash et al., 2014).

Ratwani and Trafton (2008) used eye-tracking to investigate visual search patterns of the resumption process in a simple data entry task following an interruption. The primary task required participants to place randomly generated numbers into one of fifteen different locations on a computer display following preset rules. The interruption task involved either solving math problems or performing mental rotation. Both interruption tasks impaired resumption accuracy; compared to a non-interrupted condition, individuals fixated on a location following an interruption that was further away from the correct location. However this effect was significantly larger when the interruption involved mental rotation, suggesting that the same visuo-spatial processes involved in mental rotation are important for the resumption process. Shen and Jiang (2006) also showed that an interruption involving a spatial search significantly decreased memory accuracy in a change detection search task. Both findings suggest that spatial representation may play an important role in guiding resumption after an interruption.

Despite the importance of interruptions in everyday life, the brain networks involved in interrupted performance are not known, nor have the activation of those networks been modulated. The present study used the latter strategy to better understand the neuromodulation of interruption performance. Active modulation of brain networks involved in spatial memory can provide direct evidence for the causal role of transient disruption of spatial representation in resumption performance following an interruption. There is considerable evidence that the posterior parietal cortex (PPC), and more specifically the intraparietal sulcus (IPS), is implicated in the maintenance of spatial representations (Cabeza and Nyberg, 2000; Cohen and Andersen, 2002; Jonides et al., 2005; Champod and Petrides, 2007).

**Figure 1:**
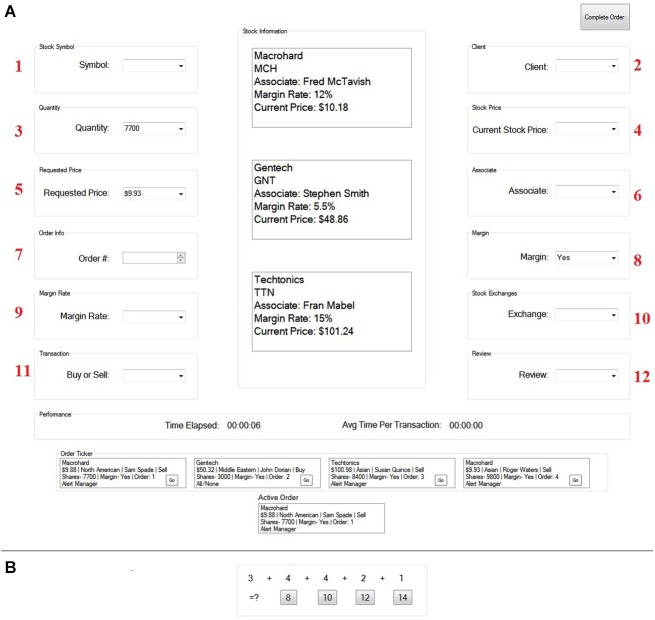
**The Financial Management Task (A) and the interruption task (B)**. The interruption task replaced the screen for the duration of each interruption.

These findings suggest that active stimulation or inhibition of the right PPC should respectively decrease or increase spatial errors during resumption after an interruption. We tested this hypothesis in the present study using transcranial Direct Current Stimulation (tDCS), which provides a method for non-invasive, bi-directional modulation of brain function (Nitsche and Paulus, 2000; Antal et al., 2001). The polarity of stimulation plays a critical role in how tDCS affects performance; typically anodal (positive) stimulation over a particular cortical site increases cortical excitability and can improve performance (Cohen Kadosh et al., 2010; Coffman et al., 2014; Parasuraman and McKinley, 2014), whereas cathodal (negative) stimulation over the cortical area inhibits excitability and may lead to decrements in task performance (Bikson et al., 2004; Coffman et al., 2014). We therefore hypothesized that anodal stimulation of the right PPC would reduce spatial errors following an interruption, whereas cathodal stimulation of the same brain region would increase errors. For the primary task, we used the *Financial Management Task*, a complex computer-based task (Trafton et al., 2011; see Figure 1) commonly used in studies of interrupted task performance and the resumption process (Trafton et al., 2003; Brumby et al., 2013). The task requires participants to store information in memory and then place that information into different locations on the computer screen, either uninterrupted or following an interruption. The interruption task required participants to solve math problems.

Our main hypothesis was that compared to a sham (placebo) group, anodal stimulation of right PPC would reduce spatial errors following an interruption, whereas cathodal stimulation would increase errors. Additionally, as a manipulation check, we also used a mental rotation task, given the causal role of the PPC in mental rotation has been previously established in a repetitive transcranial magnetic stimulation (rTMS) study (Harris and Miniussi, 2003). We hypothesized that compared to a sham (placebo) group, anodal stimulation of the right PPC would improve mental rotation accuracy, whereas cathodal stimulation would decrease accuracy. A final hypothesis, based on previous findings (e.g., Blumberg et al., in press; Tseng et al., 2012), was that lower performing individuals (those with more interruption-related errors and lower mental rotation scores) receiving anodal stimulation of the right PPC would exhibit the greatest improvements in resumption performance compared to higher performing individuals.

## Methods

### Participants

The George Mason University Institutional Review Board (IRB) approved this study. Forty-six right-handed students (*M* = 19.74 years, *SD* = 2.2, 35 females, 11 males) from George Mason University participated for course credit. One participant was excused from the study because of problems with the stimulation delivery device and the data were excluded from all analyses. Participants were randomly assigned to one of three groups: anodal stimulation (*n* = 15), cathodal stimulation (*n* = 15), and sham stimulation (*n* = 15) to the PPC. Sample size was determined based on effect sizes reported in previous modulation studies using tDCS over the PPC (e.g., Sparing et al., 2009; Stone and Tesche, 2009). Thus, the group size was set *a priori* at 15 resulting in a total sample size of 45.

### tDCS

An ActivaDose II Iontophoresis Delivery Unit was used to deliver constant current via two electrode pads housing saline-soaked sponges with an 11 cm^2^ contact area. One electrode was placed on the scalp (directly between sites CP4 and P4, identified as CPP4 of the 10–5 EEG Scalp Recording System; Oostenveld and Praamstra, 2001)—this is the same right parietal site previously found to decrease mental rotation performance in an rTMS study (Harris and Miniussi, 2003). The reference electrode was placed on the contralateral (left) upper arm. The electrodes were attached to each participant using velcro wraps. Participants received 2 mA of current for 30 min in the active stimulation group, an amount found to be safe in a number of previous studies (Coffman et al., 2014). Participants in the sham group received a 2 mA ramp up (30 s) and then immediate ramp down (6 s) of current, receiving the full 2 mA for a very short period of time (<5 s). This short stimulation duration (applied prior to the beginning of the experimental tasks) is enough to cause similar skin sensations compared to the active stimulation group, but is generally insufficient to produce lasting causal effects on cortical excitability (Coffman et al., 2014).

### Financial management task

The goal of this task was to successfully complete a client stock order as quickly and accurately as possible. To do this, participants first selected a stock order to buy or sell and then filled in twelve pieces of information relevant to that order. This information was placed, one component at a time, in one of twelve different boxes located throughout the computer screen. Importantly, participants had to place this information in order starting with the upper left box (labeled 1 in Figure 1), then the upper right box (labeled 2 in Figure 1), and so on, until all twelve pieces of information were correctly placed. If a participant went to the wrong box (i.e., made an error), the participant was unable to fill in the information. Instead, the box that the participant was supposed to go to would turn red. This indicated that an error was made and that the participant would need to place information in the red box before moving on.

Interruptions occurred randomly throughout the duration of the financial management task. The interruption task, which replaced the primary task screen, required participants to answer multiple choice addition (math) problems that were located on the bottom, center of the computer screen for the entire duration of the 15 s interruption (see Figure 1). Participants answered the problems at their own pace. Immediately following the interruption, the primary task screen reappeared and participants were able to continue the primary task. Importantly, when returning to the primary task following an interruption, all of the information that was on the screen before the interruption occurred was gone. Therefore, participants needed to remember where they left off to successfully re-engage the task without making an error (see Trafton et al., 2011 for more information about the Financial Management task).

### Mental rotation task

The Vandenberg and Kuse Mental Rotation Test, Version C (MRT-C; Peters et al., 1995; Shepard and Metzler, 1971; Vandenberg and Kuse, 1978) was used to assess mental rotation ability. This version, unlike versions A and B, and most other MRT, rotates objects around both the vertical and horizontal axes, thereby increasing the difficulty of the test. The use of this version of a mental rotation task made it less likely that individuals would be at ceiling levels of performance at baseline, thus allowing for assessment of potential improvement with anodal tDCS.

In this version, each question has one template and four possible answers (i.e., objects that when rotated match the base stimuli or objects that when rotated do not match the base stimuli). For every question, there are exactly two correct matching answers. To successfully answer the question, you must correctly identify both of the matching stimuli.

### Design and procedure

Participants first signed a consent form and were then instructed on how to complete the mental rotation task (MRT-C). Each participant completed all four practice problems with the experimenter. Following practice, participants completed the first half of the test (i.e., problems 1–12). Participants were given five minutes to the complete the problems. Participants were then trained on the Financial Management task to ensure that they were familiar with the task and minimize potential learning effects. Participants were instructed to complete both tasks (primary and interruption) as quickly and accurately as possible. The trials took approximately 75 s each to complete with interruption time removed. During baseline, participants completed 9 total trials with 27 total interruptions. Interruptions occurred randomly after the successful completion of any one box. Researchers ensured the participants were actively completing the interruption task.

Following the baseline block, the tDCS unit was set up and stimulation was applied.

The DCS Sensation Questionnaire (Scheldrup et al., 2014) was administered at three time points throughout the stimulation block. This questionnaire is used to gauge the amount of itching, heat/burning, and tingling each participant felt as a result of the stimulation; participants responded by selecting their perceived sensations on a 11-point Likert scale where 0 represented no sensation at all and 10 represented the most intense sensation imaginable. This questionnaire is required by the George Mason University IRB to ensure participants safety during the experiment; thus, the data were not analyzed *post-hoc*. Once the current value reached 2.0 mA, the DCS Sensation Questionnaire was administered. Afterwards, participants completed the stimulation block of the Financial Management task, which was identical to the design of the baseline block (i.e., 9 trials with 27 random interruptions). The DCS Sensation Questionnaire was then administered a second time. Next, participants completed the second half (i.e, problems 13–24) of the mental rotation task (MRT-C). Once complete, the final DCS Sensation Questionnaire was administered. The tDCS unit was turned off and detached from the participant. They were thanked for their participation, given a short debrief about the experiment, and then left.

### Measures

An error occurred when a participant attempted to place information in an incorrect box following an interruption; therefore, a maximum of 27 errors could be committed. Average trial completion time was computed in seconds for each participant. Performance on the interruption task was scored. Lastly, the mental rotation test (MRT-C) was scored for accuracy.

## Results

### Manipulation verification

We initially examined participants’ engagement in the interruption task. Participants successfully answered 83% (*SD* = 5.1, range: 74–96%) of the multiple choice math problems, suggesting they were actively engaged in the interruption task and not rehearsing the primary task.

To determine if interruptions affected performance on the primary task, we compared the number of errors a participant made when completing the task without interruptions (*M* = 0.47, *SD* = 0.66) to the number of errors a participant made following an interruption (*M* = 12.71, S*D* = 2.81) in the baseline trials. A paired samples *t*-test confirmed that the interruptions negatively affected performance, *t*_(44)_ = 27.51, *p* < 0.001, *d* = 4.10.

**Figure 2:**
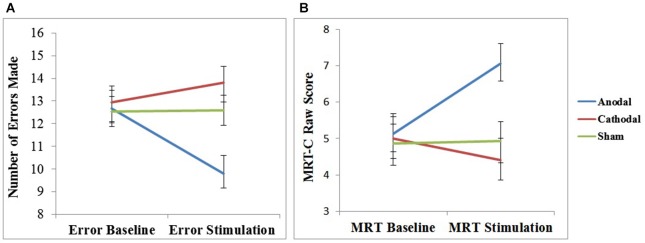
**The mean number of errors made (fewer errors represent improved performance) following an interruption (A) and the mean raw scores (higher scores represent improved performance) on the mental rotation task (B) during baseline and stimulation blocks for the three stimulation group (anodal, cathodal, and sham)**. Plotted with standard errors of the mean.

Before determining if tDCS affected performance, we needed to ensure that no baseline differences existed between the three stimulation groups (anodal, cathodal, and sham). A one-way analysis of variance (ANOVA) revealed no differences existed in the number of errors made during the baseline trials between groups, *F*_(2,42)_ = 0.076, *p* > 0.250, $\eta_{\mathrm{partial}}^{2}$ = 0.004, see Figure 2A. A separate one-way ANOVA of the MRT-C revealed no differences existed in baseline scores (i.e., problems 1–12) between groups as well, *F*_(2,42)_ = 0.056, *p* > 0.250, $\eta_{\mathrm{partial}}^{2}$ = 0.003, see Figure 2B.

### Interruption-related errors

A mixed-design ANOVA was performed to determine whether tDCS affected the number of errors participants made following an interruption. The within-subject factor was block (baseline and stimulation) and the between-subject factor was stimulation group (anodal, cathodal, and sham). Levene’s test indicated equal error variances in both the baseline (*F* = 0.09, *p* > 0.250) and stimulation (*F* = 0.84, *p* > 0.250) data. There was a significant main effect of block, *F*_(1,42)_ = 8.68, *p* = 0.005, $\eta_{\mathrm{partial}}^{2}$ = 0.17 and a significant interaction between block and stimulation group, *F*_(2,42)_ = 26.93, *p* < 0.001, $\eta_{\mathrm{partial}}^{2}$ = 0.56, see Figure 2A.

Tests of simple main effects using a Bonferroni correction (*α* = 0.05) within the anodal stimulation group revealed that significant differences existed between the number of errors in baseline (*M* = 12.67, 95% CI [11.17, 14.16]) and stimulation (*M* = 9.8, 95% CI [8.35, 11.25]), *t*_(14)_ = 7.56, *p* < 0.001, *d* = 1.95. Tests of simple main effects using a Bonferroni correction (*α* = 0.05) within the cathodal stimulation group revealed that significant differences existed between the number of errors made at baseline (*M* = 12.93, 95% CI [11.44, 14.43]) and during stimulation (*M* = 13.8, 95% CI [12.35, 15.25]), *t*_(14)_ = 2.29, *p* = 0.027, *d* = 0.59. No differences existed within the sham group (*p* > 0.250). On average anodal stimulation resulted in three fewer spatial errors (i.e., 23% reduction), whereas cathodal stimulation increased spatial errors by one (i.e., 7% increase), and sham did not change performance.

Tests of simple main effects using a Bonferroni correction (*α* = 0.05) within the stimulation block revealed that significant differences existed between the number of errors committed in the anodal stimulation group (*M* = 9.8, 95% CI [8.35, 11.25]) compared to both the cathodal stimulation group (*M* = 13.8, 95% CI [12.35, 15.25]), *t*_(28)_ = 3.94, *p* = 0.001, *d* = 1.49) and sham stimulation group (*M* = 12.6, 95% CI [11.15, 14.05], *t*_(28)_ = 2.76, *p* = 0.026, *d* = 1.04), but not between the cathodal and sham stimulation groups (*p* > 0.250; see Figure 2A). On average individuals receiving anodal stimulation made three fewer errors (i.e., 22% reduction) in the stimulation block compared to individuals in the sham stimulation group and four fewer errors (i.e., 29% reduction) compared to individuals in the cathodal stimulation group.

**Figure 3:**
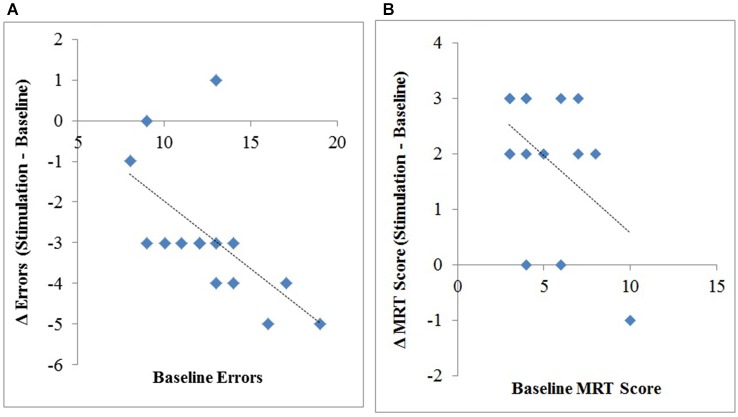
**Change (stimulation–baseline) in interruption-related errors (A) and MRT scores (B) plotted against the number of errors (A) and mean mental rotation scores (B) at baseline**.

We also correlated the number of errors each participant in the anodal stimulation group made at baseline to their change in errors (stimulation minus baseline), revealing a significant correlation, *r*_(14)_ = −0.61, *p* = 0.016, *R*^2^ = 0.37. This suggests that individuals with worse initial performance (i.e., more errors in baseline) benefitted the most from anodal stimulation, see Figure 3A.

### Mental rotation task (MRT-C)

A mixed-design ANOVA was performed to determine whether tDCS affected performance on the mental rotation task. The within-subject factor was block (baseline and stimulation) and the between-subject factor was stimulation group (anodal, cathodal, and sham). Levene’s test indicated equal error variances in both the baseline (*F* = 0.218, *p* > 0.250) and stimulation (*F* = 0.074, *p* > 0.250) data. There was a significant main effect of block, *F*_(1,42)_ 8.32, *p* = 0.006, $\eta_{\mathrm{partial}}^{2}$ = 0.17 and a significant interaction between block and stimulation group, *F*_(2,42)_ = 21.96, *p* < 0.001, $\eta_{\mathrm{partial}}^{2}$ = 0.51, see Figure 2B.

Tests of simple main effects using a Bonferroni correction (*α* = 0.05) within the anodal stimulation group revealed that significant differences existed between mental rotation accuracy during baseline (*M* = 5.133, 95% CI [4.00, 6.27]) and in stimulation (*M* = 7.07, 95% CI [5.96, 8.17]), *t*_(14)_ = 6.90, *p* < 0.001, *d* = 1.78. Tests of simple main using a Bonferroni correction (*α* = 0.05) effects within the cathodal stimulation group revealed that significant differences existed between mental rotation accuracy at baseline (*M* = 5.00, 95% CI [3.86, 6.14]) and during stimulation (*M* = 4.40, 95% CI [3.29, 5.51]), *t*_(14)_ = 2.14, *p* =.038, *d* = 0.55. No differences existed within the sham group (*p* > 0.250). On average participants in the anodal stimulation group improved mental rotation score by two (i.e., 27% improvement), cathodal stimulation decreased mental rotation score by half a point (i.e., 12% reduction), and sham did not change performance.

Tests of simple main effects using a Bonferroni correction (*α* = 0.05) within the stimulation block revealed that significant differences existed between MRT-C scores in the anodal stimulation group (*M* = 7.07, 95% CI [5.96, 8.17]) compared to both the cathodal stimulation group (*M* = 4.40, 95% CI [3.29, 5.51], *t*_(28)_ = 3.44, *p* = 0.004, *d* = 0.89) and sham stimulation group (*M* = 4.93, 95% CI [3.83, 6.04], *t*_(28)_ = 2.75, *p* = 0.026, *d* = 0.71). Scores in the cathodal stimulation group were not significantly different from sham (*p* > 0.250; see Figure 2B). On average individuals receiving anodal stimulation scored two points higher (i.e., 30% improvement) on the mental rotation task compared to individuals in the sham stimulation group and two and a half points higher (i.e., 38% improvement) than individuals in the cathodal stimulation group.

Additionally, we correlated each participants MRT-C score in the anodal stimulation group at baseline to their change in MRT-C score (stimulation minus baseline), revealing a significant correlation, *r*_(14)_ = −.47, *p* = 0.04, *R*^2^ = 0.22, however this effect is largely driven by one participant given the relatively low amount of variability (*s*^2^ = 4.5) in MRT-C scores at baseline, see Figure 3B.

### Completion time

To determine whether tDCS affected average trial completion time, a mixed-design ANOVA was performed to determine whether tDCS affected average trial completion time across all three groups, with the within-subject factor being block (baseline and stimulation) and the between-subject factor being stimulation group (anodal, cathodal, and sham). Levene’s test indicated equal error variances in both the baseline (*F* = 0.859, *p* > 0.250) and stimulation (*F* = 0.331, *p* > 0.250) data. There was a main effect of block, *F*_(1,42)_ = 7.69, *p* = 0.008, $\eta_{\mathrm{partial}}^{2}$ = 0.16 and a significant interaction between block and stimulation group, *F*_(2,42)_ = 7.169, *p* = 0.002, $\eta_{\mathrm{partial}}^{2}$ = 0.25.

Tests of simple main effects using a Bonferroni correction (*α* = 0.05) within the anodal stimulation group revealed that a significant difference existed (*p* < 0.001) between average trial completion time in baseline (*M* = 77.07s, 95% CI [72.69, 81.45]) and average trial completion time in stimulation (*M* = 72.13s, 95% CI [67.93, 76.34]). No differences existed between baseline and stimulation average completion time in the cathodal or sham stimulation groups (*p* > 0.250 for both). That is, individuals in the anodal stimulation group completed the task more quickly while stimulated compared to baseline. This may not be a surprise as these same individuals made fewer errors and making an error would result in more time spent on that trial.

### Mental rotation and errors

**Figure 4:**
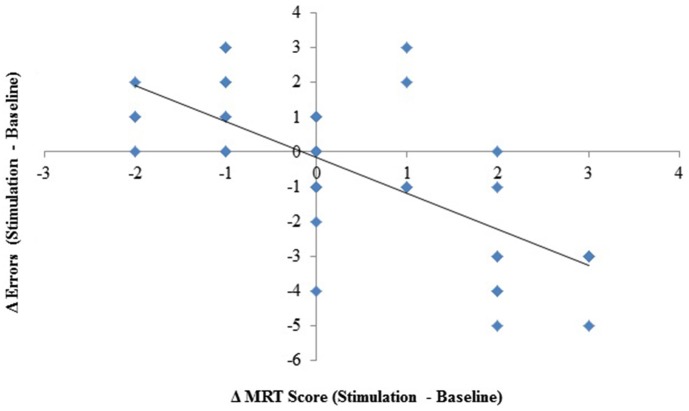
**Change (stimulation–baseline) in interruption-related errors plotted against change in mental rotation scores**.

Given that the processes that guide resumption after an interruption may recruit the same neural substrates as mental rotation, it is likely that changes in one (mental rotation) may be reflected in changes in the other (resumption process, i.e., errors). To examine the extent to which they are related, we correlated the difference scores (stimulation minus baseline) for both measures, including all three stimulation groups. The analysis revealed a significant correlation, *r*_(45)_ = −.72, *p* < 0.001, *R*^2^ = 0.52, see Figure 4. The magnitudes of the changes in performance for each measure were significantly related.

## Discussion

The brain networks involved in the interruption process are not known and the neuromodulation of those networks has not been previously been examined. Given that spatial representations aid in the resumption process after an interruption (Ratwani and Trafton, 2008) and that the right PPC is activated during the maintenance of spatial representations (Cabeza and Nyberg, 2000; Cohen and Andersen, 2002), we hypothesized that anodal stimulation of this region would reduce the number of errors by enhancing memory for spatial information. Additionally, we hypothesized that cathodal stimulation applied to the PPC would increase the number of errors by diminishing spatial representation ability. The results supported these hypotheses: anodal stimulation of right PPC significantly reduced the number of interruption-related errors while increasing mental rotation accuracy, whereas cathodal stimulation of the same region had the opposite effects, and sham stimulation did not affect either performance measure.

To our knowledge, this is the first demonstration of bi-directional effects of activation and inhibition of PPC on spatial errors following interruptions and on mental rotation performance. The results provide evidence for a causal role for the PPC in the maintenance of spatial representations during interrupted task performance. We also found that the magnitude of the changes in interruption-related errors with tDCS was significantly related to changes in mental rotation performance, as measured by the MRT-C. Specifically, individuals who improved in mental rotation accuracy exhibited a reduction in the number of interruption errors to a similar degree. This finding supports the idea that spatial representation ability, as assessed using the MRT-C, guides resumption after an interruption. The findings are unlikely to reflect a placebo effect given that sham stimulation did not affect performance.

Additionally, we found that lower performing individuals at baseline testing, measured by both the number of interruption errors and MRT-C, showed the greatest improvements in performance following anodal stimulation of PPC. This result suggests that individual differences in baseline ability may modulate the behavioral effects of tDCS. Such “cognitive equalizing” due to tDCS was also previously reported in a study of change detection (Tseng et al., 2012). Our finding that lower-performing individuals showed greater benefits of tDCS than higher-performing ones diminishes concerns that tDCS and other non-invasive brain stimulation techniques may widen or exacerbate ability differences in the population, thereby leading to greater social inequality (Cohen Kadosh et al., 2012).

During stimulation, performance in both the interruption and mental rotation tasks was significantly greater in the anodal group than in the cathodal and sham groups. However, whereas cathodal stimulation significantly reduced performance on both tasks compared to baseline, the cathodal and sham groups did not differ significantly following stimulation. Some other previous tDCS studies have also found that effects of cathodal stimulation are often less pronounced than anodal effects (Fregni et al., 2005; Tseng et al., 2012; Coffman et al., 2014). Another limitation in the present study is that although we designed the tDCS montage to target the IPS based on current modeling (Datta et al., 2009) and previous literature (Harris and Miniussi, 2003), the relatively non-focal nature of tDCS means that other brain regions could also have been stimulated and could have played a role in the effects. In addition, each participant received only one type of stimulation; therefore it is possible that other individual differences that were not assessed in this study could have been responsible for the differential effects of anodal and cathodal stimulation on interruption errors and mental rotation performance. Additionally, math problems were included as the interruption task in the present study even though mental rotation has been shown to interfere with the resumption process to a greater extent (Ratwani and Trafton, 2008). Given that tDCS produced significant effects in resumption performance in the less interfering task (math problems), potentially greater effects may be found with mental rotation. Finally, many tasks that can be interrupted exist (e.g., giving verbal commands) that may not benefit from anodal stimulation of the PPC when interrupted because the task is not spatial in nature. Therefore, we cannot generalize our results to all tasks and forms of interruption.

This is the first study to show how noninvasive brain stimulation can reduce human error following interruptions. Interruptions are unavoidable, and while many only cause delays or reduce efficiency, they can also lead to serious errors (Westbrook et al., 2010; Prakash et al., 2014). Importantly, tDCS offers a safe, inexpensive, and easy to administer method to reduce errors during the resumption process. This study offers bi-directional causal support for the role of PPC in mental rotation ability and in the resumption process. Important issues that need to be addressed in future research include retention of tDCS-induced benefits on interruption performance and their transfer to other tasks (Parasuraman and McKinley, 2014).

## Conflict of interest statement

The authors declare that the research was conducted in the absence of any commercial or financial relationships that could be construed as a potential conflict of interest.
